# Antioxidative Properties of Crude Polysaccharides from *Inonotus obliquus*

**DOI:** 10.3390/ijms13079194

**Published:** 2012-07-23

**Authors:** Haibo Mu, Amin Zhang, Wuxia Zhang, Guoting Cui, Shunchun Wang, Jinyou Duan

**Affiliations:** 1 College of Science, Northwest A&F University, Yangling, Shaanxi 712100, China; E-Mails: mhb1025@sina.com (H.M.); wmyzyqyybfk@126.com (A.Z.); wuxia200758@163.com (W.Z.); moohybo@yahoo.cn (G.C.); 2 Shanghai University of Traditional Chinese Medicine, Shanghai 201203, China

**Keywords:** *Inonotus obliquus*, polysaccharide, antioxidant, reactive oxygen species

## Abstract

The mushroom *Inonotus obliquus* has been widely used as a folk medicine in Russia, Poland and most of the Baltic countries. In this study, water-soluble and alkali-soluble crude polysaccharides (IOW and IOA) were isolated from *I. obliquus*, and the carbohydrate-rich fractions IOW-1 and IOA-1 were obtained respectively after deproteination and depigmentation. Their contents, such as neutral carbohydrate, uronic acid and protein, were measured. Their antioxidant properties against chemicals-induced reactive species (ROS) including 1,1′-Diphenyl-2-picrylhydrazyl (DPPH) radical, hydroxyl radical and superoxide anion radical, as well as their protective effects on H_2_O_2_-induced PC12 cell death were investigated. Results showed that *I. obliquus* polysaccharides can scavenge all ROS tested above in a dose-dependent manner. IOA and its product IOA-1 could rescue PC12 cell viability from 38.6% to 79.8% and 83.0% at a concentration of 20μg/mL. Similarly, IOW and its product IOW-1 at the same dose, can also increase cell viability to 84.9% and 88.6% respectively. The antioxidative activities of water-soluble and alkali-soluble polysaccharide constituents from *I. obliquus* might contribute to diverse medicinal and nutritional values of this mushroom.

## 1. Introduction

The mushroom *Inonotus obliquus* (*I. obliquus*), belonging to the Hymenochaetaceae family of Basidiomycetes, is a black parasitic fungus that grows on living trunks of the mature birch and is mainly found at latitudes of 45°N–50°N [1,2]. As early as in the sixteenth century, *I. obliquus* was used as an effective folk medicine in Russia and Northern Europe to treat several human malicious tumors such as breast cancer, liver cancer, uterine cancer and gastric cancer and other diseases like hypertension and diabetes [3], with little toxic side effects [4]. Recently, many polyphenolic compounds, triterpenoids [5], and steroids, such as lanosterol, inotodiol [6], trametenolic acids, and ergosterol peroxide [7] from this fungus have been identified and their biological activities, including hypoglycemic [1], hepato-protective [8] have been demonstrated. Polysaccharides are one of the main components of *I. obliquus* and they have been shown to exhibit many biological activities including anti-tumor [9], antioxidant, hypoglycemic and immune-stimulating effects [10,11].

Reactive oxygen species (ROS) is defined as chemically active products generated by partial reduction in oxygen, including free radicals such as hydroxyl and superoxide as well as non-free radicals such as hydrogen peroxide. In living organisms, prolonged elevated levels of ROS can cause intense oxidative stress, which is considered to play a very important role in the pathogenesis of several degenerative diseases [12] and pathological effects such as causing DNA damage, carcinogenesis and cellular degeneration related to aging [13]. To help defend against oxidative damage, synthetic antioxidants such as butylated hydroxytoluene (BHT), butylated hydroxyanisole (BHA) and tert-butylated hydroxyquinone (TBHQ) are commonly used. However, there has been growing concern over their safety and toxicity [14,15]. Thus, natural antioxidants, for example, fucoidan [16], chitosan [17], tea polysaccharide [18], Lycium barbarum polysaccharide [19], which can scavenge ROS *in vitro* and *in vivo* attract more attention.

Herein, we reported antioxidative effects of water-soluble and alkali-soluble crude polysaccharides from *I. obliquus* on chemicals-induced ROS, as well as the protective effects on H_2_O_2_-induced PC12 cell death.

## 2. Results and Discussion

### 2.1. Major Chemical Contents

As shown in Table 1, the water-soluble and alkali-soluble crude polysaccharides IOW and IOA from *I. obliquus* contain both neutral carbohydrates and proteins with small amount of uronic acid (<5%). After deproteinization and depigmentation procedures, as expected, both IOW-1 and IOA-1 were predominantly composed of neutral carbohydrates (>50%). The rigorous deproteinization process through the sevag method didn’t get rid of protein in IOW and IOA completely, implying that some proteins in IOW-1 and IOA-1 might be existed as polysaccharide-protein conjugates.

### 2.2. Antioxidative Effects on Chemicals-Induced ROS

The antioxidative activities of antioxidants have been attributed to various mechanisms, including prevention of chain reaction, binding of transition metal ion catalysts, decomposition of peroxides, prevention of continued proton abstraction, reductive capacity and radical scavenging [20]. Due to the complexity of the oxidation-antioxidation processes, one test is normally not enough to evaluate precisely the antioxidant activity of the potential antioxidant [21]. Therefore, the following four assays were applied to evaluate the antioxidant capacity of the crude polysaccharides from *I. obliquus.*

#### 2.2.1. DPPH Radical Scavenging Capability

**Figure 1:**
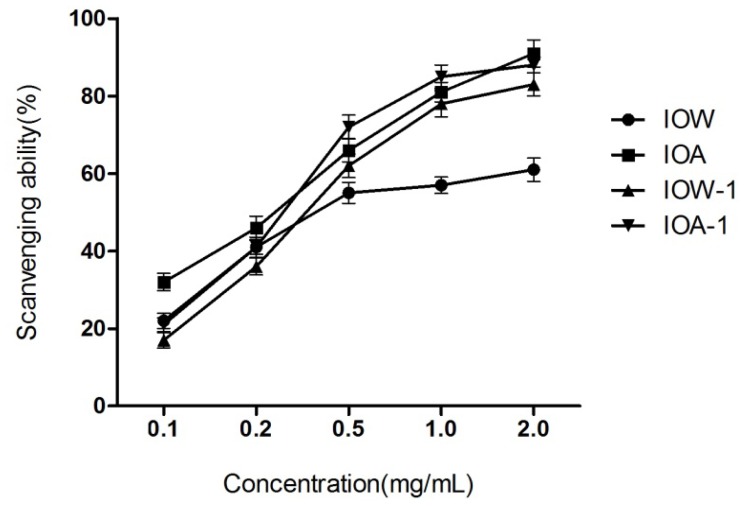
Scavenging ability on DPPH radicals.

DPPH is a well-known radical which demonstrates a strong absorption band centered at about 520 nm, and it becomes colorless or pale yellow when neutralized. DPPH radical is scavenged by antioxidants through the donation of proton forming the reduced DPPH, and is commonly used to evaluate the radical scavenging capacity of antioxidants [22]. The scavenging activities of IOW, IOW-1, IOA and IOA-1 against the DPPH radical were shown in Figure 1. As observed, all four samples could neutralize the DPPH radical, and this activity was concentration-dependent at the range of 0.1–2.0 mg/mL. In contrast to that in IOA, the existence of protein and pigment in IOW significantly decreased its DPPH radical scavenging activity at a high concentration (>0.5 mg/mL).

#### 2.2.2. Hydroxyl Radical Scavenging Activity

**Figure 2:**
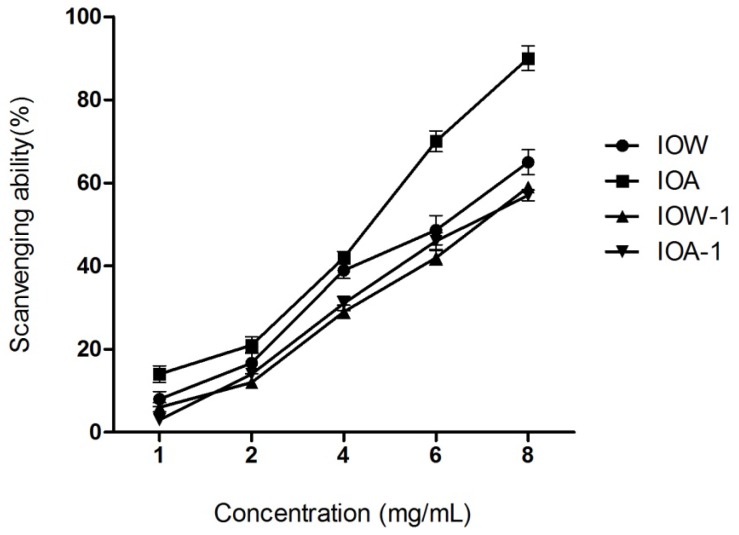
Scavenging ability on hydroxyl radicals.

The hydroxyl radical, more likely to be produced *in vivo*, is considered to be the most reactive and poisonous free radical in living organisms. This radical can damage virtually all types of macromolecules including carbohydrates, lipids, proteins, and nucleic acids [23]. As for carbohydrates, this radical can abstract hydrogen atoms at all ring C–H bonds of aldoses, uronic acids, and other sites on carbohydrates except C-2 of *N*-acetyl hexosamine [24]. The hydroxyl radical in the cells can easily cross cell membranes at specific sites and cause tissue damage and cell death. Thus, removing hydroxyl radical is very important for the protection of living systems [25]. As shown in Figure 2, all four samples exhibited obvious scavenging activity on hydroxyl radical in a concentration-dependent pattern. Deproteinization and depigmentation of IOW, but not IOA decreased its hydroxyl radical scavenging effects at the range of 4–8 mg/mL.

#### 2.2.3. Superoxide Anion Scavenging Capacity

**Figure 3:**
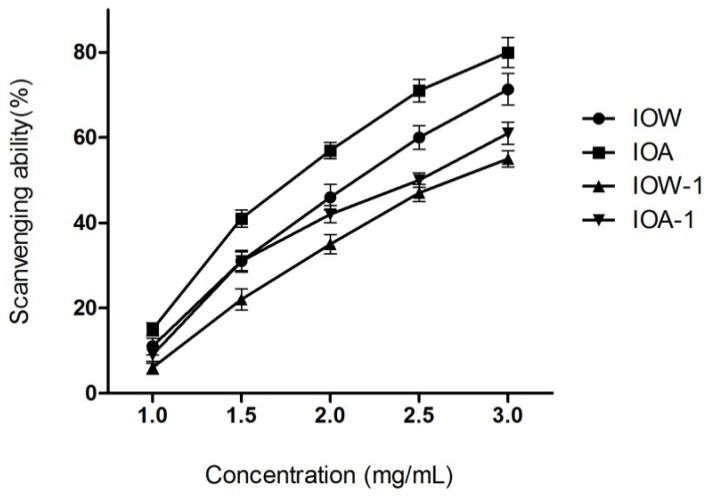
Scavenging ability on superoxide radicals.

Superoxide anion (O_2_^−^), known to be produced *in vivo*, plays important roles in the formation of ROS such as hydrogen peroxide, hydroxyl radical and singlet oxygen, which induces oxidative damage in lipids, proteins and DNA [26]. All four samples elicited the O_2_^−^ scavenging activity which followed a dose dependent manner (Figure 3). Furthermore, the protein and pigment in both IOW and IOA increased this scavenging capacity.

#### 2.2.4. EC_50_ Values

The results above from three different assays indicated that four polysaccharide fractions tested exhibited a noticeable capacity of scavenging DPPH radicals, hydroxyl radicals and superoxide anion. The antioxidant properties assayed herein were summarized in Table 2 and the results were normalized and expressed as EC_50_ values (mg/mL).

### 2.3. Ferric Reducing Power Assay

**Figure 4:**
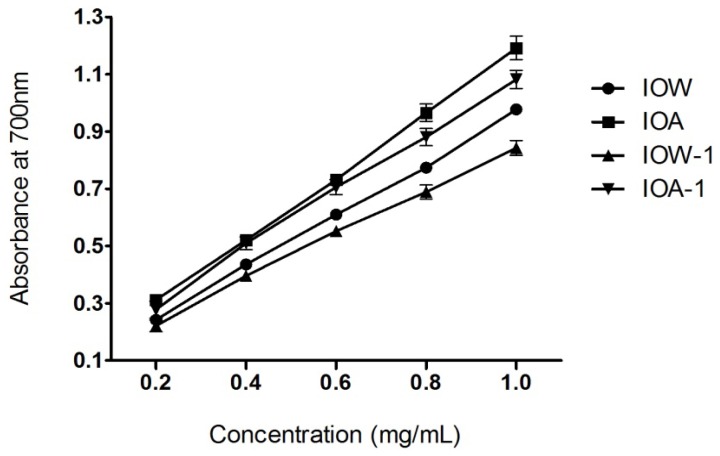
Reducing power.

The FRAP (ferric-reducing antioxidant power) assay treats antioxidants contained in the samples as reductants in a redox-linked colormetric reaction, and the value reflects the reducing power of the antioxidants [27]. The assay is also commonly used for the routine analysis of single antioxidants and total antioxidant activity of plant extracts by measuring Fe^3+^-Fe^2+^ conversion. The dose-response curve for the reducing activity of four samples was shown in Figure 4. In addition, the alkali-soluble crude polysaccharide IOA demonstrated a stronger reducing activity than the water soluble crude polysaccharide IOW. This trend still remained after the deproteinization and depigmentation procedure.

### 2.4. Protective Effects on H_2_O_2_-Induced PC12 Cell Death

H_2_O_2_ is able to penetrate biological membranes, and plays a radical forming role as an intermediate in the production of more reactive ROS molecules including formation of hydroxyl radical via oxidation of transition metals, and hypochlorous acid by the action of myeloperoxidase, an enzyme present in the phagosomes of neutrophils [28], H_2_O_2_ has been used in many studies to trigger cell apoptosis [29,30].

**Figure 5:**
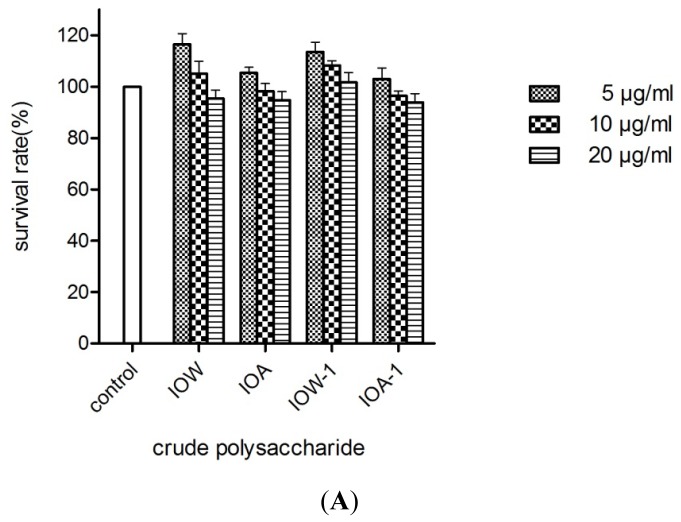

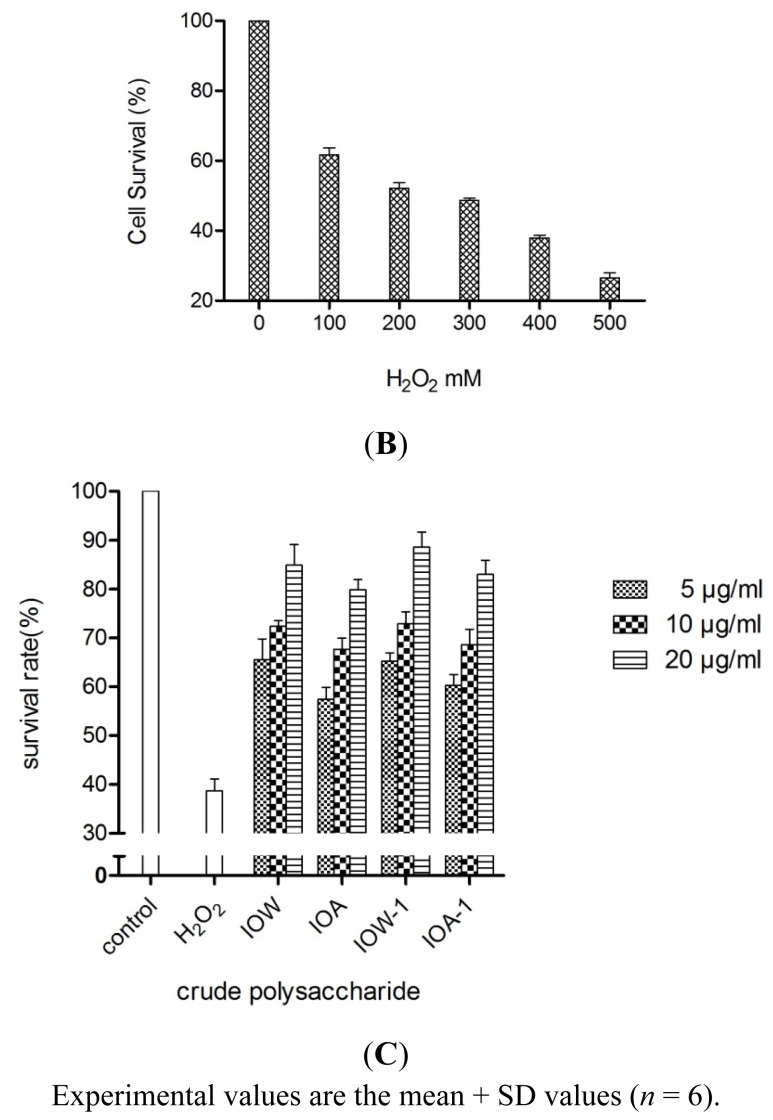
(**A**) Viability percentages of PC12 cells treated with crude polysaccharides; (**B**) MTT reduction assay of H_2_O_2_ cytotoxicity; (**C**) Cell viability of crude polysaccharides on H_2_O_2_-induced death in PC12 cells.

Antioxidants could prevent PC12 cell death through the suppression of H_2_O_2_-induced ROS formation [30], the regulation of the endogenous oxidant–antioxidant balance [31], and the alterations in a variety of intracellular signaling pathways such as PI3K/Akt pathway [32], *etc.* PC12 cells is derived from a pheochromocytoma of the rat adrenal medulla and widely used as a model cell line to assess the protective effects of an antioxidant on H_2_O_2_-induced cell death. Four samples, IOW, IOW-1, IOA or IOA-1 at the dose of 5–20 μg/mL had no significant toxic effects on PC12 cells (Figure 5A). The MTT assay showed that 100–500 μM of H_2_O_2_ could result in the PC12 cell death dramatically (Figure 5B). For instance, the incubation of PC12 cells with 400 μM H_2_O_2_ for 6 h resulted in a cell viability rate of 38.6% compared to the control (Figure 5C). However, when pretreating the cells with three different concentrations (5, 10, 20 μg/mL) of IOW, IOW-1, IOA or IOA-1, the cell viability was significantly increased by 19–50% (Figure 5C).

## 3. Materials and Methods

### 3.1. Materials and Chemicals

The sclerotia of *I. obliquus* were purchased from Heilongjiang Beiqishen High-Tech Health Products Co., Ltd, Helongjiang Province, China. Dulbecco’s modified Eagle’s medium (DMEM) was purchased from Gibco Ltd. (Grand Island, NY, USA) and fetal bovine serum (FBS) was from Hyclone (Logan, UT, USA). 1,1′-Diphenyl-2-picrylhydrazyl (DPPH) and MTT (3-(4,5-dimethylthiazol-2-yl)-2,5-diphenyl tetrazolium bromide) were obtained from Sigma Co.(St. Louis, MO, USA). All other chemicals and reagents were of analytical grade as available.

### 3.2. Preparation of Crude Polysaccharides from *I. obliquus*

**Figure 6:**
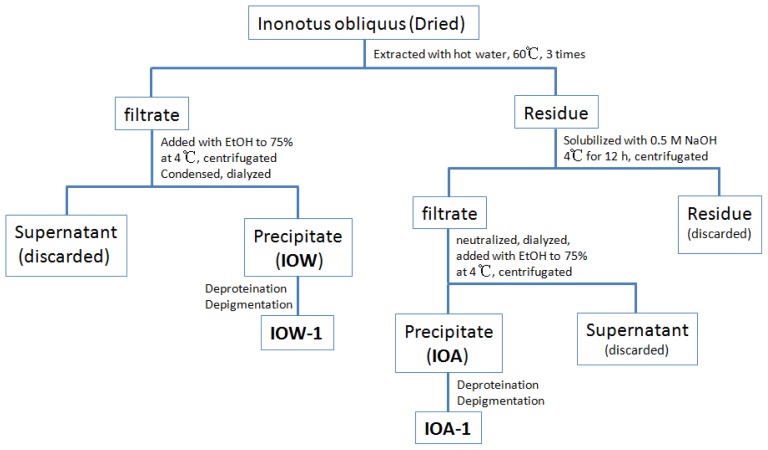
Preparation of crude polysaccharides from *I. obliquus*.

As shown in Figure 6, the powder of *I. obliquus* was extracted three times with water at 60 °C for 4 h. The extract was centrifuged at 4000 r/min for 20 min, and the supernatant was combined, dialyzed (Spectra/Por RC dialysis membrane, MW cutoff 3500 Da), and then concentrated. Three volumes of 95% ethanol were added to precipitate polysaccharides at 4 °C overnight. The precipitate was collected after centrifugation at 4000 r/min for 10min, washed with absolute acetone and then air dried (35 °C) to yield the water soluble polysaccharide designed as IOW. The residue after water extraction was extracted with 0.5 M NaOH at 4 °C for 12 h. After the supernatant was neutralized with HCl, the alkali-soluble polysaccharide IOA was finally obtained according to the similar procedures as described above.

The polysaccharides (IOW and IOA) were dealt with deproteinization and depigmentation, yielding IOW-1 and IOA-1. Sevag reagent as a chemical method was used in the deproteinization and hydrogen peroxide in depigmentation. In brief, IOW and IOA were treated by Sevag reagent (the ratio of IOW and IOA to Sevag reagent was 3:1) CHCl_3_−nBuOH (v/v = 4:1) seven times to deproteinize.

### 3.3. Properties of Crude Polysaccharides

#### 3.3.1. Determination of Total Neutral Carbohydrate Contents

The carbohydrate contents were determined with a slightly modified phenol-sulphuric acid method [33]. Briefly, 400 μL of sample solution, 400 μL 5% phenol, and 2mL of concentrated sulphuric acid were mixed and shaken. After the mixture was kept at room temperature for 30 min, the absorbance was measured at 490 nm. The total carbohydrate content was calculated with D-glucose as standard.

#### 3.3.2. Determination of Uronic Acid Contents

This was determined according to the method of Blumenkrantz [34] using d-galacturonic acid as standard.

#### 3.3.3. Determination of Protein Contents

The total protein content was measured by the method of Bradford [35], with bovine serum albumin as standard.

### 3.4. Antioxidant Activity Assay

#### 3.4.1. DPPH Radical Scavenging Assay

The radical scavenging effects of samples on DPPH radical were estimated as described [36]. Briefly, sample solution (2 mL) at different concentrations was added to equivalent aliquot DPPH (0.1 mM) in 95% ethanol. The reaction solution was shaken vigorously and incubated at room temperature for 30 min, and the absorbance at 517 nm was measured. Ascorbic acid was used as a positive control.

The DPPH radical scavenging percenta (P) was calculated as follows:

$$\text{P}(\%)=[1-\frac{\text{Abs}(\text{sample})-\text{Abs}(\text{control})}{\text{Abs}(\text{blank})}]\times 100$$

where the control solution contains equivalent distilled water instead of the DPPH solution, while distilled water instead of sample was used for the blank. All tests were performed in triplicate and the mean of Abs was used in the equation above.

#### 3.4.2. Hydroxyl Radical Scavenging Assay

This was determined according to the literature [37]. 0.5 mL of salicylic acid-ethanol solution (9.1 mM), 0.5 mL of sample solution at different concentrations, 0.5 mL of FeSO_4_ solution (9.1 mM) and 3.0 mL of distilled water were successively mixed in a tube. The reaction was initiated by the addition of 3.0 mL H_2_O_2_ (8.8 mM) to the mixture above, and the absorbance at 510 nm was read. The hydroxyl radical scavenging activity percentage (P) was calculated as follows:

$$\text{P}(\%)=[1-\frac{\text{Abs}(\text{sample})-\text{Abs}(\text{control})}{\text{Abs}(\text{blank})}]\times 100$$

where the distilled water instead of H_2_O_2_ was used for the control, while distilled water instead of sample was used for the blank. All tests were performed in triplicate and the mean of Abs was used in the equation above.

#### 3.4.3. Superoxide Anion Scavenging Capacity

Pyrogallol (1,2,3-benzenetriol) can autoxidize rapidly, especially in alkaline solution, and produce superoxide anion [38]. The scavenging capabilities of all samples were determined according to the method of Marklund [39]. Different concentrations of sample solution (0.1 mL) were mixed with 2.5 mL of Tris-HCl buffer (pH 7.8, 50 mM) containing 1 mM EDTA. After 20 min at room temperature, 0.4 mL of pyrogallol (7 mM) was added and the mixture was shaken rapidly. The absorbance of the mixture was measured at 420 nm per 30 s for 4 min, and the slope was calculated as k (abs/min). The scavenging activity was calculated as follows:

$$\text{P}(\%)=1-\frac{\text{k}(\text{sample})-\text{k}(\text{blank})}{\text{k}(\text{control})}]\times 100$$

where the mixture without samples was the control and the mixture without pyrogallol was the blank. Ascorbic acid was used as the positive control.

#### 3.4.4. Ferric Reducing Power Assay

The reducing power was determined as described [40] with a slight modification. 0.5 mL of sample solutions at different concentration were mixed with 0.5 mL of phosphate buffer (0.2 M, pH 6.6) and 0.5 mL of potassium ferricyanide (1%, w/v). The mixture was incubated at 50 °C for 20 min, and then 0.5 mL of trichloroacetic acid (10%, w/v) was added. After centrifuged at 3000 rpm for 10 min, the upper layer (1 mL) was mixed with deionized water (1 mL) and ferric chloride (0.2 mL, 0.1%), and the absorbance was measured at 700 nm.

### 3.5. Cell Cytotoxicity and Protective Effects on H_2_O_2_-Induced PC12 Cell Death

These were evaluated *in vitro* using the MTT assay [41]. PC12 cells were cultured in DMEM (High Glucose) medium supplemented with 10% fetal bovine serum (FBS), 100 U/mL penicillin and 100 μg/mL streptomycin. To check the cell cytotoxicity, cell suspensions were seeded in 96-well plates (1 × 10^5^ /well), and incubated at 37 °C for 12 h, and the sample was added. After 6 h, 20 μL of the MTT stock solution (5 mg/mL) were added into each well and the plate was further incubated for 4 h. Finally, the medium was removed and DMSO (200 μL) was added to each well to dissolve the formazan. After 10 min, the absorbance was measured at 570 nm in a microtitre plate reader. For protective assay, samples were added to the cultured cells and incubated for 30 min before the addition of H_2_O_2_ (final concentration 400 μM, 6 h), other procedures were same as above. Assays were performed in quadruplet wells for each sample. Data were expressed as the percent of cell viability compared with control (mean ± SD).

## 4. Conclusions

In this study, the water-soluble and alkali-soluble polysaccharides, IOW and IOA were isolated from *I. obliquus.* After deproteination and depigmatation, the carbohydrate-rich fractions IOW-1 and IOA-1 were obtained. Their chemical compositions were determined by colorimetric methods, and their antioxidative properties were evaluated by chemicals-induced ROS assays including radical-scavenging activity (DPPH, hydroxyl radical superoxide anion) and ferric-reducing antioxidant power assay. In addition, their protective effects on H_2_O_2_-induced PC12 cell death were explored. Results indicated that *I. obliquus* polysaccharides possess multiple radical scavenging activities in a dose-dependent manner and protect PC12 cells from H_2_O_2_-induced death. These antioxidant activities might account, at least in part, for the pharmaceutical effects of *I. obliquus.*

## Figures and Tables

**Table 1:** Major chemical content of the alkaline and water extracts.

	IOA	IOA-1	IOW	IOW-1
Carbohydrate content (wt.%)	22.28 ± 0.31	50.13 ± 0.47	21.23 ± 0.42	61.21 ± 0.29
Protein content (wt.%)	10.60 ± 0.92	6.28 ± 0.84	14.07 ± 1.05	7.69 ± 0.81
Uronic acid content (wt.%)	3.79 ± 0.43	4.12 ± 0.51	4.60 ± 0.36	4.51 ± 0.20

Each value is expressed as mean ± SD (*n* = 3).

**Table 2:** EC_50_ values of the extracts from *I. obliquus* in radical scavenging ability.

EC_50_ (mg/mL)				

	IOW	IOA	IOW-1	IOA-1
DPPH radicals	0.56 ± 0.06	0.23 ± 0.03	0.37 ± 0.05	0.27 ± 0.04
Hydroxyl radicals	5.66 ± 0.34	3.44 ± 0.31	7.00 ± 0.22	6.45 ± 0.17
Superoxide anion	2.12 ± 0.12	1.80 ± 0.08	2.62 ± 0.07	2.39 ± 0.09

EC_50_ value: The effective concentration at which the antioxidant activity was 50%; Each value is expressed as mean ± SD (*n* = 3).
